# The Effect of Ingesting Alginate-Encapsulated Carbohydrates and Branched-Chain Amino Acids During Exercise on Performance, Gastrointestinal Symptoms, and Dental Health in Athletes

**DOI:** 10.3390/nu16244412

**Published:** 2024-12-23

**Authors:** Lotte L. K. Nielsen, Max Norman Tandrup Lambert, Jørgen Jensen, Per Bendix Jeppesen

**Affiliations:** 1 Department of Clinical Medicine, Aarhus University Hospital, Aarhus University, Palle Juul-Jensens Boulevard 165, 8200 Aarhus, Denmarkmntl@clin.au.dk (M.N.T.L.); 2 Department of Physical Performance, Norwegian School of Sports Sciences, 0863 Oslo, Norway

**Keywords:** hydrogels, alginate, carbohydrates, BCAA, athletic performance, TTE, recovery, gastrointestinal comfort, dental health

## Abstract

Background: This study aimed to compare the effects of a carbohydrate (CHO) hydrogel with (ALG-CP) or without (ALG-C) branched-chain amino acids, and a CHO-only non-hydrogel (CON), on cycling performance. The hydrogels, encapsulated in an alginate matrix, are designed to control CHO release, potentially optimising absorption, increasing substrate utilisation, and reducing gastrointestinal distress as well as carious lesions. Methods: In a randomised, double-blinded, crossover trial, 10 trained male cyclists/triathletes completed three experimental days separated by ~6 days. During the experimental days, participants completed a standardised 2 h cycling bout (EX1), followed by a time-to-exhaustion (TTE) performance test at W_75%_. Supplements were ingested during EX1. Results: Participants cycled ~8.8 (29.6%) and ~5.4 (29.1%) minutes longer during TTE with ALG-CP compared to ALG-C and CON, respectively. TTE was 65.28 ± 2.8 min with ALG-CP, 56.46 ± 10.92 min with ALG-C, and 59.89 ± 11.89 min with CON. Heart rate (HR) was lower during EX1 with ALG-CP (*p* = 0.03), and insulin levels increased more significantly during the first 45 min with ALG-CP. Plasma glucose and glucagon levels remained consistent across supplements, although glucagon was higher with ALG-CP before TTE. Post-exercise myoglobin levels were lower with ALG-CP compared to ALG-C (*p* = 0.02), indicating reduced muscle damage. Conclusions: While ALG-CP improved performance duration compared to ALG-C and CON, the difference did not reach statistical significance. Additionally, there was a lower HR during the cycling session, alongside a significantly lower level of myoglobin with ALG-CP. These findings suggest that ALG-CP may offer advantages in cycling performance and recovery.

## 1. Introduction

It is common practice among athletes to ingest energy gels or sugary beverages during prolonged exercise. The intake of carbohydrates during prolonged endurance exercise has been demonstrated to maintain or improve performance [1,2,3]. The ergogenic effects of CHO are likely related to the maintenance of plasma glucose, keeping high rates of CHO oxidation and delaying fatigue; however the exact mechanisms are still unclear [1,2,4,5,6,7,8]. In line with this, current guidelines highlight a high CHO availability during prolonged strenuous exercise lasting 2.5 h or more, with an intake of up to 90 g CHO/hour [9].

Although amino acids (AA) are not considered a major fuel source during endurance exercise, an increase in protein degradation and leucine oxidation has been demonstrated during exercise, which may be further enhanced under low-glycogen conditions [10,11,12,13]. Co-ingestion of protein and CHO has previously been shown to enhance performance after recovery periods ranging between 5 and 18 h compared to CHO-only [14,15,16,17]. Additionally, co-ingesting protein and CHO during exercise has been reported to improve athletic performance by reducing muscle damage [12,18,19,20,21,22,23]. However, this is not a universal finding [18,24,25,26,27,28].

Cycling is often characterised as a long-duration and high-intensity sport [29]. Most cycling races involve both relatively steady-state periods and bursts of high-intensity efforts, including sprinting [29]. Therefore, the present study was designed such that participants cycled for 2 h at varying intensities (47–80% W_max_) while ingesting CHO with or without the addition of BCAA before we tested the performance. Even though frequent consumption of CHO is recommended to improve prolonged endurance performance, excess intake of CHO during exercise may also increase the risk of gastrointestinal (GI) distress [30,31,32,33,34,35]. Accordingly, some prior studies have reported symptoms of GI distress (e.g., nausea, flatulence, vomiting) during prolonged endurance exercise while consuming CHO-rich beverages, meals, and gels [30,31,35,36,37]. Although still unclear, GI distress induced by excess CHO intake during exercise has been proposed to be caused by gastric distension due to a reduction in gastric emptying (GE) and by incomplete absorption with excess CHO intake [31,33,34,35,38,39]. Notably, the occurrence of GI distress has been associated with impaired performance in athletes [30,34,36,37,40]. Moreover, endurance athletes are considered at risk of developing dental caries due to the intake of CHO in large quantities [41,42,43]. The development of dental caries is a multifactorial and dynamic process in which tooth demineralisation occurs as a result of organic acids (e.g., lactic acid) being produced by bacterial (e.g., Streptococcus mutans) degradation of mainly sugars [44,45].

A new alginate-encapsulated supplement (ALG) has been developed as a novel way to provide CHOs. The concept of encapsulating CHO in alginate-based hydrogels may be beneficial in terms of allowing the consumption of CHO in high concentration, increasing the GE rate and the absorption of CHO, as well as reducing GI symptoms and the risk of dental caries by acting as a barrier preventing dental exposure to CHOs, which could lead to improved performance and health benefits [43,46,47,48,49]. Alginate is a polymer extracted from seaweed, which has been applied in drug delivery to encapsulate proteins [50,51,52]. Prior studies investigating the effect of CHO-containing hydrogels provided a CHO beverage containing alginate and pectin, which forms a hydrogel when reaching the low pH in the stomach [46,47,49,53,54]. In contrast to these studies [43,46,47,49,53,54], we provided CHOs that were already encapsulated in alginate-based hydrogels prior to ingestion [51].

The primary aim of this study was to compare the effect of a CHO hydrogel with or without BCAA and a CHO-matched non-hydrogel supplement ingested during a 2 h standardised cycling bout on time-to-exhaustion (TTE) performance immediately afterwards. Additionally, the present study compares the effect of delivering CHOs by two different delivery systems (i.e., hydrogel vs. non-hydrogel) on performance, gastrointestinal symptoms, and dental health. Secondary endpoints included assessment of plasma glucose level, hormones (insulin, glucagon), and muscle damage markers.

## 2. Materials and Methods

### 2.1. Participant Characteristics

Ten healthy male participants (mean ± SD: age: 33.9 ± 11.9 years; height: 181.9 ± 6.2 cm; body weight: 73.9 ± 7.7 kg; VO_2max_: 63.5 ± 7 mL·min^−1^·kg^−1^; Watt_max_: 421.1 ± 44.4 watts) volunteered for this study. The included participants were moderate to well-trained male cyclists, triathletes, or mountain bikers. The inclusion criteria consisted of men between 18 and 50 years of age, who were accustomed to cycling training with a VO_2max_ ≥ 50 mL·min^−1^·kg^−1^. Exclusion criteria consisted of metabolic diseases that relates to CHO metabolism, usage of medications/dietary supplements or following a low-CHO diet, which might influence CHO metabolism during cycling and/or recovery. The protocol was approved by the Regional Ethics Committee of Central Denmark (Journal no.: 1-10-72-154-21. Date: 17 May 2021). The study was conducted in accordance with the Declaration of Helsinki. Prior to enrolment, all participants gave their informed written consent after an oral and written briefing by the research team, who provided the subjects with information about the study (clin.trial.no.: NCT06165393).

### 2.2. Experimental Design

The study was conducted as a double-blinded randomised cross-over control study. Subjects completed one session of physiological measures (i.e., preliminary test) approximately a week before the experimental trials, which was followed by three experimental intervention days separated by approximately six days. The experimental day consisted of a standardised cycling bout of 2 h (EX1) followed by a TTE performance test and a subsequent 2 h recovery period afterwards. During EX1 the participants ingested either: encapsulated CHO and BCAA (ALG-CP), encapsulated CHO-only (ALG-C), and CHO-only, which was not encapsulated (CON). Furthermore, they consumed a standardised dinner and breakfast prior to each trial day.

### 2.3. Preliminary Test

The subjects performed a VO_2max_ test on a cycle ergometer approximately a week before the experimental trial. The participants were introduced to the VO_2max_ protocol before initiating the test. They started cycling at 150 watts for 5 min, thereafter the load increased with 25 W every minute until the subjects could not hold the cadence within +/−5 RPM. The cadence was self-selected between 80 and 95 (+/−5 RPM), which kept throughout the whole study. Criteria for a successful VO_2max_ measurement included a failure for VO2 to increase with increasing workload and a respiratory exchange ratio (RER) > 1.10. The preliminary test served to determine their individual VO_2max_ in order to ensure that the participants adhered to the inclusion criteria. Watt_max_ was calculated as workload of the last stage completed + [(25 W/60 s) × seconds at the final stage] [16,55]. After the VO_2max_ test the participants had a small break before completing the familiarisation cycling bout, which consisted of a shortened version of the experimental exercise protocol.

### 2.4. Experimental Trials

Subjects arrived at the clinic in the morning approximately 2 h following consumption of a standardised breakfast. They were weighed and then fitted with a heart rate monitor (Polar H10 heart rate sensor, Polar Electro, Kempele, Finland). Baseline urine, blood, and saliva samples were collected before initiating the EX1 session, and the first portion of the supplement was provided before starting the EX1 as well.

The exercise protocol consisted of the following: (1) 3 × 8 min interval cycling at 47%, 52%, and 58% W_max_, (2) for the next 60 min the intensity alternated every 6 min between 72% and 55% W_max_, and (3) two sets of 4 × 2 min intervals at 80% W_max_. The intervals and sets were separated by 2 min and 5 min cycling at 55% W_max_, respectively. This was followed by (4) 5 min cycling at 50% W_max_, and then (5) TTE performance test at 75% W_max_. The exercise protocol was inspired and modified from prior studies [21,53]. In this study, exhaustion was defined as subjects not being able to maintain the pedal frequency (±5 rpm) at the given workload despite three verbal encouragements [15,55].

Heart rate (HR) and Rate of Perceived Exhaustion (RPE) (i.e., 6–20 Borg Scale) was measured during EX1 and TTE. A questionnaire on experienced GI symptoms was provided after the performance test using RedCap (version 8.10.20). Participants rated their perceived GI symptoms on the 0–10 Visual Analogue Scale (VAS, [40]), and symptoms included nausea, flatulence, and diarrhoea. Water was ingested ad libitum during exercise. All testing was completed in thermoneutral conditions (21.9 ± 1.3 °C) with uniform cooling provided by a pedestal fan. Subjects were asked to refrain from exercise the day before the experimental trial. They were provided with a standardised dinner and breakfast to be consumed before the experimental day. The dinner contained: CHO: ~1.17 g/kg, PRO: ~1.04 g/kg, FAT: ~0.18 g/kg and energy: ~49.8 kJ/kg. The breakfast contained CHO: ~0.85 g/kg, PRO: ~0.22 g/kg, FAT: ~0.21 g/kg, and energy: ~27.03 kJ/kg. The intervention supplements consisted of ALG-CP (CHO and BCAA hydrogel), ALG-C (CHO hydrogel), and CON (CHO non-hydrogel) (Jens Møller Products ApS, Herning, Denmark). The CHO source was maltodextrin/glucose (1:1), and the BCAAs consisted of 49% leucine, 26% isoleucine, and 25% valine (Eurofins, Vejen, Denmark). Both ALG-CP and ALG-C were provided as calcium-alginate hydrogels. All three supplements were provided in similar, opaque packets, similar to energy-gel packets already on the market. Supplements were matched for CHO content and were provided every 15 min during the EX1 bout, providing the participants with 75 g CHO/h and 8 g BCAA/h with ALG-CP. The supplements were provided in random order using a REDCap Randomization Module. The allocation was blinded to investigators and participants.

### 2.5. Biological Samples

Venous blood samples were collected from a Teflon catheter, which was inserted into an antecubital vein, in order to collect samples during the exercise bout. Blood samples were collected in 4 mL Li-Hep tubes (BD, Plymouth, UK) and 2.0 mL and 6.0 mL EDTA tubes (BD, Plymouth, UK), placed on ice and centrifuged for 10 min at 4 °C (2500× *g*). Plasma was then pipetted into 2 mL Eppendorf tubes (SARSTEDT, Nümbrecht, Germany) and stored at −80 °C until subsequent analysis. Plasma samples were analysed for: glucose, insulin, glucagon, FFA, creatine kinase (CK), lactate dehydrogenase (LDH), myoglobin, and carbamide (P-Carbamide).

Glucose. Principle of analysis was enzymatic reaction measured with absorption photometry. The kit used was Atellica CH Glucose Hexokinase_3 (ref: 11097592). Glucose concentration was analysed on Siemens Atellica CH 930 (Siemens Healthineers, Erlangen, Germany).

Insulin. Concentration of insulin was analysed on Roche cobas e601 (Roche, Rotkreuz, Germany), using Elecsys Insulin kit (ref: 12017547122).

Glucagon. Analyses was determined using Mercodia ELISA kit (10-1271-01) (Mercodia AB, Uppsala, Sweden).

FFA. Analyses was on PerkinElmer Victor x5, 2030 multilabel Reader, using an NEFA-HR(2) R1 (434-91795) and R2 (436-91995) Assay kit (FujiFilm Wako Chemicals Europe, Neuss, Germany).

Creatine Kinase. CK concentration was analysed using Atellica CH Creatine Kinase kit (CK_L, ref: 11097640) (Siemens Healthineers, Erlangen, Germany) on Siemens Atellica CH 930 (Siemens Healthineers, Erlangen, Germany).

LDH. Principle of analysis was enzymatic reaction measured with absorption photometry. Analysis was conducted on Siemens Atellica CH 930 (Siemens Healthineers, Erlangen, Germany), using an Atellica CH Lactate Dehydrogenase L-P (LDLP) kit (ref: 11097594).

P-Carbamide. Principle of analysis was enzymatic reaction measured with absorption photometry. Analysis was conducted on Siemens Atellica CH 930 (Siemens Healthineers, Erlangen, Germany), using Atellica CH Urea Nitrogen (UN_c) kit (ref: 11097593).

Myoglobin. Concentration of myoglobin was analysed on Siemens Atellica IM (Siemens Healthineers, Germany), using Atellica CH Lactate Dehydrogenase L-P (LDLP) (ref: 11097594).

Urine samples were collected in urine sample cups at baseline and post-TTE. A 12 mL sample was frozen and stored at −80 °C.

U-Carbamide was analysed on Siemens Atellica CH 930, using Atellica CH Uric Acid (UA) kit (ref 11097608) (Siemens Healthineers, Erlangen, Germany).

U-Creatinine was analysed on Siemens Atellica CH 930, using Atellica CH Enzymatic Creatinine_2 (ECre_2) kit (ref 11097533) (Siemens Healthineers, Erlangen, Germany).

**Figure 1:**
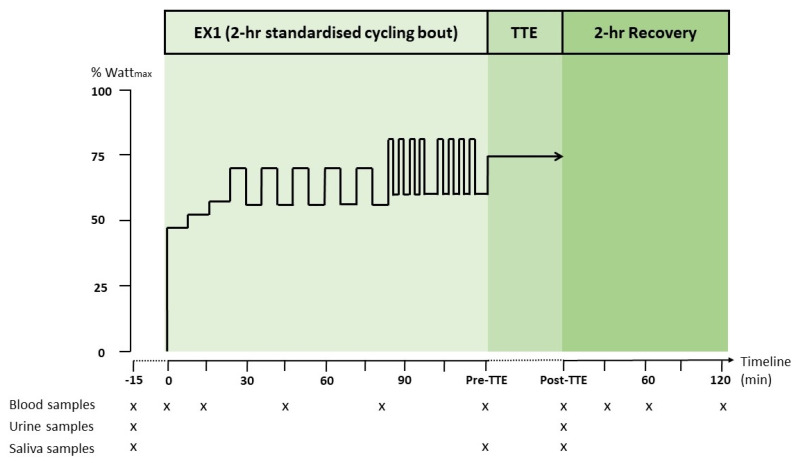
Overview of experimental clinical trial.

Saliva samples were collected at baseline, prior to TTE and post-TTE (Figure 1). The pH value of the saliva samples was measured immediately after collecting the sample by using a pH meter (PHM92, LAB pH METER, Radiometer Denmark A/S, Copenhagen, Denmark). This procedure was in a previously study determined in collaboration with the Department of Dentistry and Oral Health, Aarhus University [55].

### 2.6. Statistics

All data were assessed for normal distribution by visual inspection of QQ plots. Plasma baseline values were calculated as the mean of two plasma samples collected before initiating the EX1 bout/ingesting supplement with 15 min between sampling. Data that were not normally distributed were log transformed and tested for normal distribution, these consists of plasma insulin, glucagon, FFA, myoglobin, CK, U-Creatinine. Repeated measures (RM) ANOVA was used for data analysis of the performance test (i.e., TTE) as well as for area under the curve (AUC) analyses of plasma glucose, insulin, glucagon, and FFA performed for exercise and recovery phases (bar charts are only shown when data were significant). A two-way RM ANOVA (treatment × time) was used for analysis of physiological parameters (i.e., plasma, saliva, and urine samples) with a Geisser–Greenhouse correction. Bonferroni post hoc test was used in case of significant interactions. Differences were considered significant when *p* ≤ 0.05. The Friedman test was used to assess any statistically differences in perceived GI symptoms. Data denoted with (¤) were statistically analysed with log data and presented with raw data. Statistical analyses were performed using GraphPad Prism 10.1.2. All following data are presented as mean ± SEM, if otherwise not specified.

## 3. Results

### 3.1. Performance

**Figure 2:**
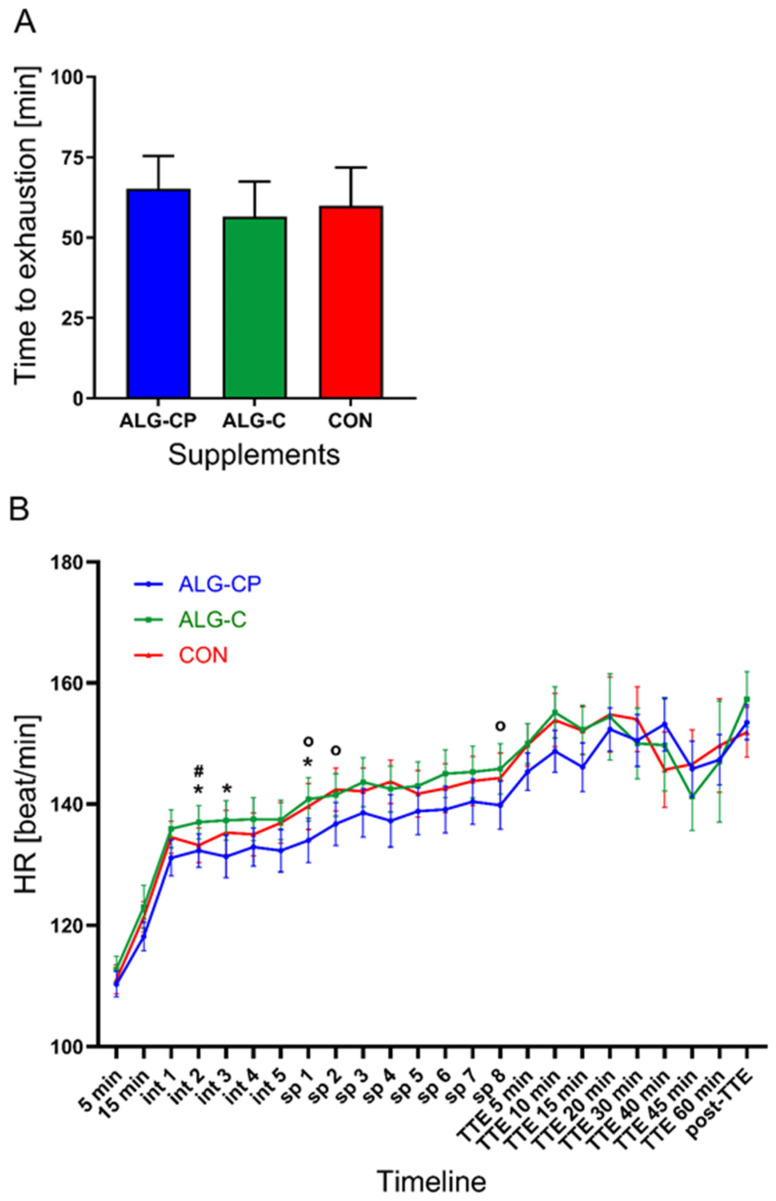
Presenting (**A**) time-to-exhaustion performance test in minutes, and (**B**) Heart rate (HR, beat per min.) throughout the exercise session. Data are mean ± SEM. N = 10 participants. Int: Interval; sp: sprint; TTE: time-to-exhaustion. *: significant differences between ALG-CP vs. ALG-C. #: significant differences between ALG-C vs. CON. O: significant differences between ALG-CP vs. CON.

The performance results are presented in Figure 2. Mean time-to-exhaustion during the TTE cycling test was not statistically different between ALG-CP (65.28 ± 2.8 min), ALG-C (56.46 ± 10.92 min), and CON (59.89 ± 11.89 min) (*Anova*: *p* = 0.4). Although, with ALG-CP, the participants’ cycling time was ~8.8 min longer on average compared to ALG-C and ~5.4 min longer compared to CON. There were no statistical differences in the amount of water ingested during the exercise (*p* > 0.05). There was a main effect of treatment in HR during exercise (*p* = 0.03, Figure 2). The post hoc test revealed lower HR with ALG-CP compared to both CHO conditions at specific timepoints during EX1. No difference in HR was found between supplements during TTE (*p* = 0.12). There was a tendency for lower HR at TTE-15 min after ALG-CP compared to CON (*p* = 0.053). There were no differences in HR during the exercise protocol in test order (*p* = 0.8). Moreover, a time effect was observed for RPE, which increased during the exercise session (*p* < 0.0001). There were no differences in RPE between supplements (*p* = 0.2) nor in regard to test order (*p* = 0.5).

### 3.2. Physiological Responses

#### 3.2.1. Plasma Glucose

**Figure 3:**
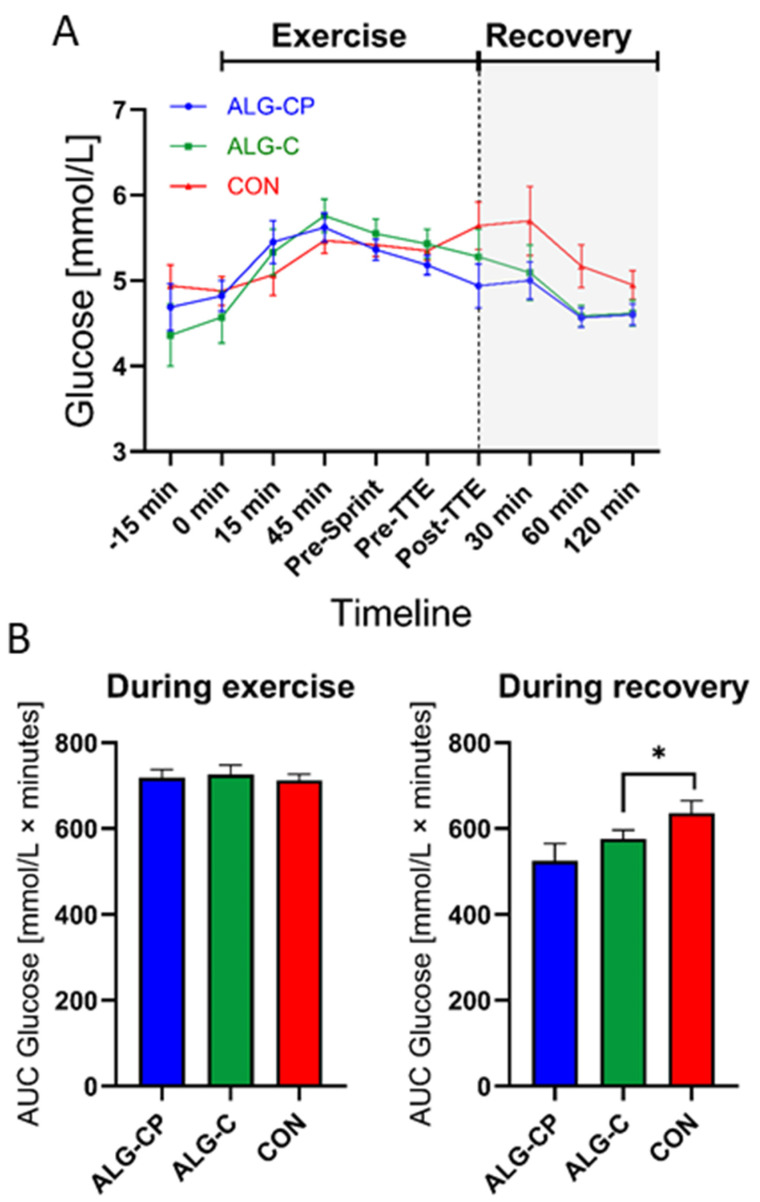
Plasma glucose during the clinical trial (**A**). Comparing ALG-CP, ALG-C, and CON. AUC during exercise is provided from t = −15 min to pre-TTE (**B**). During recovery AUC is given from t = post-TTE to 120 min. Data are mean ± SEM. * *p* ≤ 0.05.

During exercise we observed similar plasma glucose concentrations between supplements with a main effect of time (*p* = 0.03) and of time × treatment (*p* = 0.01) (Figure 3). The post hoc test showed a significant difference between ALG-CP and ALG-C at pre-TTE (*p* = 0.048). During the 2 h recovery phase, there was a main effect of time (*p* = 0.02) with a decline in plasma glucose levels with CON from post-TTE to 120 min (*p* = 0.01, Figure 3). During recovery, the AUC was significantly higher for glucose with CON compared to ALG-C (*p* = 0.05). No difference in the AUC during exercise was found.

#### 3.2.2. Plasma Insulin

**Figure 4:**
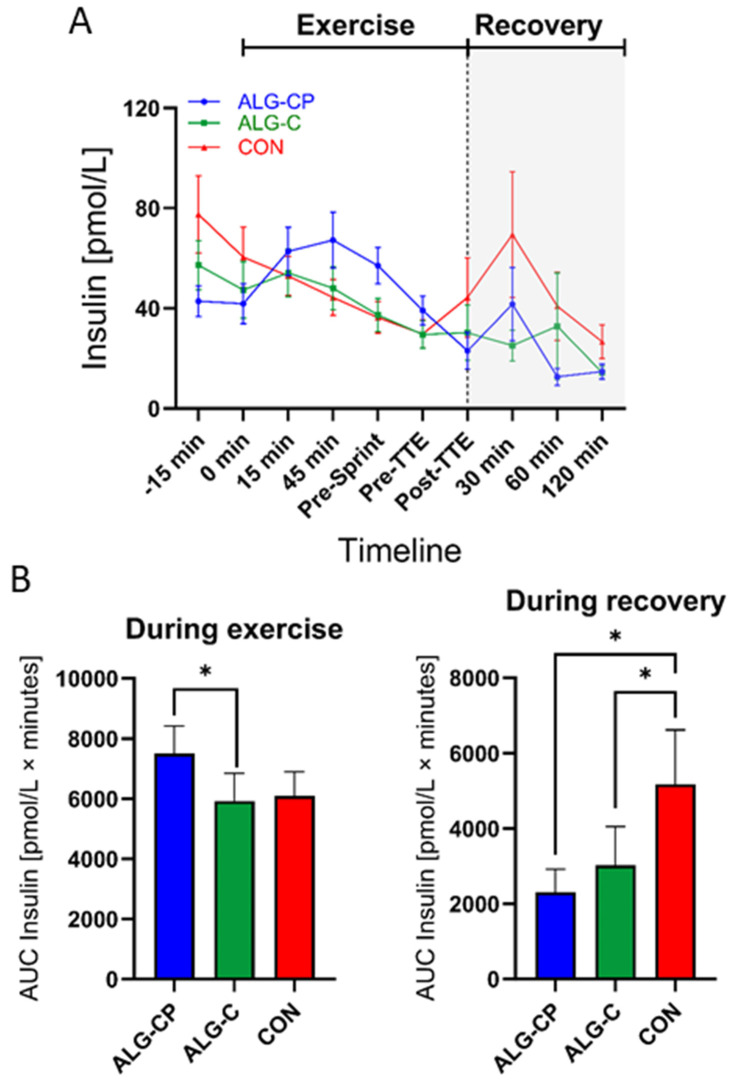
Plasma insulin during the clinical trial (**A**). Comparing ALG-CP, ALG-C, and CON. AUC during exercise is provided from t = −15 min to pre-TTE (**B**). During recovery, AUC is given from t = post-TTE to 120 min. Data are mean ± SEM. (¤). * *p* ≤ 0.05.

During exercise there was a main effect of time (*p* = 0.01) and time × treatment (*p* = 0.006) for insulin levels (Figure 4). With the intake of ALG-CP during exercise we observed an increase in plasma insulin levels from baseline to 45 min, which thereafter decreased. The course of the curve for plasma insulin was similar for ALG-C and CON from baseline to pre-TTE with decreasing levels of plasma insulin. Thereafter, plasma insulin increased with CON from post-TTE to the 30 min time-point of recovery. Post hoc test revealed a higher level of insulin at baseline with CON compared to ALG-CP (*p* = 0.02). There was a higher insulin level with ALG-CP during exercise at 45 min (compared to ALG-C, *p* = 0.05) with a tendency for higher levels compared to CON (*p* = 0.06). At pre-sprint, insulin levels were higher with ALG-CP compared to ALG-C and CON (*p* = 0.008 and *p* = 0.03, respectively), and there was a tendency at pre-TTE for higher insulin levels with ALG-CP vs. CON (*p* = 0.06). In addition, AUC levels were significantly higher for insulin with ALG-CP compared to ALG-C (*p* = 0.05) during exercise and tended to be higher after ALG-CP compared to CON (*p* = 0.06). There was a main treatment effect during recovery (*p* = 0.03). Post hoc test revealed a higher insulin level with CON during recovery at 30 min (compared to ALG-C, *p* = 0.04) and a tendency at 120 min of recovery (compared to ALG-C, *p* = 0.06). In addition, AUC was higher with CON compared to ALG-CP (*p* = 0.03) and ALG-C (*p* = 0.05) during recovery.

#### 3.2.3. Plasma Glucagon

**Figure 5:**
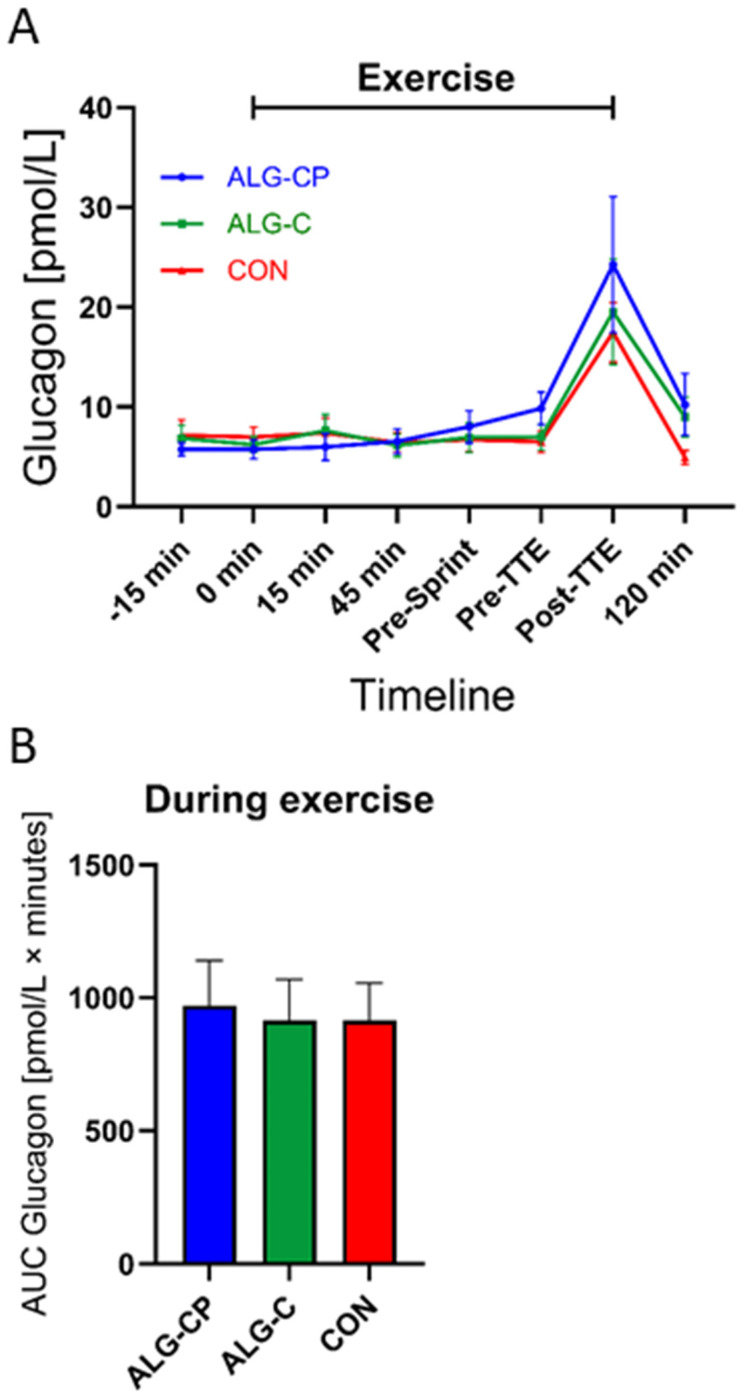
Plasma glucagon during the clinical trial (**A**). Comparing ALG-CP, ALG-C, and CON. AUC during exercise is provided from t = −15 min to pre-TTE (**B**). Data are mean ± SEM. (¤).

Plasma glucagon concentrations were similar between supplements from baseline to pre-sprint (Figure 5). There was a main effect of time (*p* < 0.0001) and time × treatment (*p* = 0.03) with higher glucagon levels at pre-TTE when ingesting ALG-CP compared to ALG-C and CON (*p* = 0.0007 and *p* = 0.006, respectively). At post-TTE, the glucagon levels increased similarly with all three supplements and decreased again at 120 min of recovery, where the levels of glucagon were lower with the intake of CON compared to ALG-CP and to ALG-C (*p* = 0.02 and *p* = 0.03, respectively). During exercise, there were no significant differences in AUC between supplements.

#### 3.2.4. Plasma FFA

**Figure 6:**
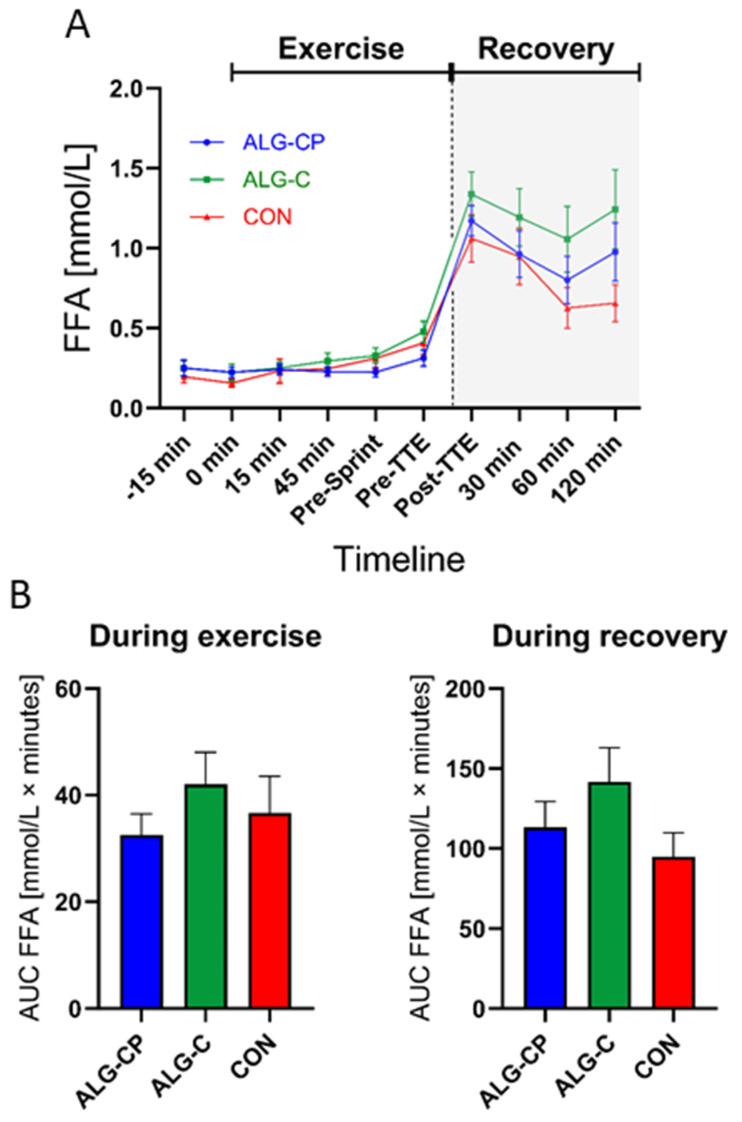
Plasma FFA during the clinical trial (**A**). Comparing ALG-CP, ALG-C, and CON. AUC during exercise is provided from t = −15 min to pre-TTE (**B**). During recovery, AUC is given from t = post-TTE to 120 min. Data are mean ± SEM. (¤).

Plasma FFA levels were constant at similar levels with all three supplements during the first hour of cycling (Figure 6). During exercise there was a main effect of time (*p* < 0.0001) and time × treatment (*p* = 0.03). The post hoc test revealed differences at baseline (*p* = 0.05) between ALG-CP and CON at pre-sprint (*p* = 0.004) and at pre-TTE (*p* = 0.002) between ALG-CP and ALG-C. During recovery, there was a main effect of time (*p* = 0.02) and treatment (*p* = 0.03). Post hoc revealed a difference at post-TTE (*p* = 0.05) and at 120 min (*p* = 0.03) with higher FFA levels after ALG-C compared to CON. RM one-way ANOVA showed no differences in AUC data between supplements during exercise and recovery.

#### 3.2.5. Muscle Damage Parameters and Protein Degradation

**Figure 7:**
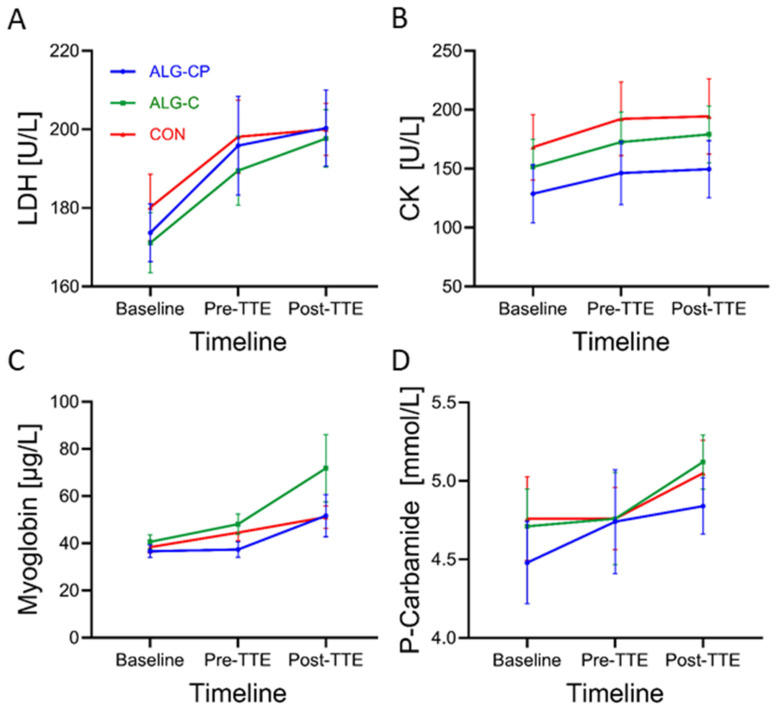
Time-dependent concentrations of (**A**) LDH, (**B**) CK (¤), (**C**) Myoglobin (¤), and (**D**) P-Carbamide during the clinical trials. Data are mean ± SEM.

There was a main effect of time for LDH (*p* < 0.0001). The post hoc test revealed increasing levels of LDH from baseline to pre-TTE, with ALG-CP and a tendency for ALG-C (*p* = 0.02 and *p*= 0.06, respectively). In addition, LDH levels increased from baseline to post-TTE for both hydrogels (ALG-CP: *p* = 0.002 and ALG-C: *p* = 0.002) (Figure 7). There were no differences between supplements. A main time effect was observed for CK (*p* < 0.0001) for all three supplements. Levels of CK increased significantly from baseline to pre-TTE and from baseline to post-TTE for all three supplements. There was a significant main time effect for P-Carbamide (*p* = 0.008) with increasing levels from baseline to post-TTE with ALG-C (*p* = 0.03) and a tendency for increased levels with CON from pre-TTE to post-TTE (*p* = 0.054). There was a significant increase in plasma myoglobin from baseline to post-TTE (*p* = 0.04) and from pre-TTE to post-TTE (*p* = 0.03) when ingesting CON. There was a significant treatment effect for myoglobin at pre-TTE with reduced levels of myoglobin when ingesting ALG-CP compared to ALG-C (*p* = 0.02).

#### 3.2.6. Urine and Saliva

**Figure 8:**
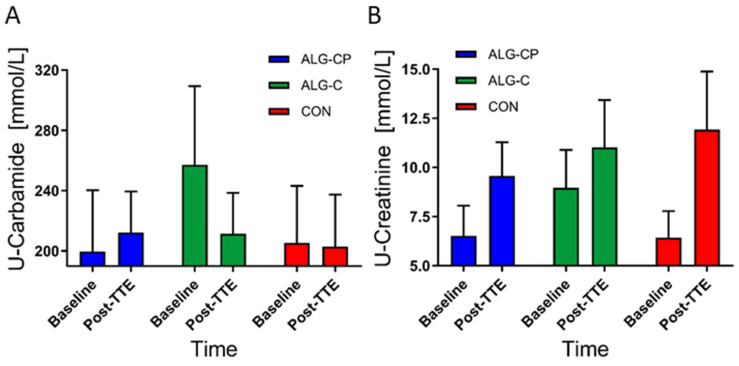
Levels of (**A**) U-Carbamide and (**B**) U-Creatinine (¤) at baseline and post-TTE. Data are mean ± SEM.

**Figure 9:**
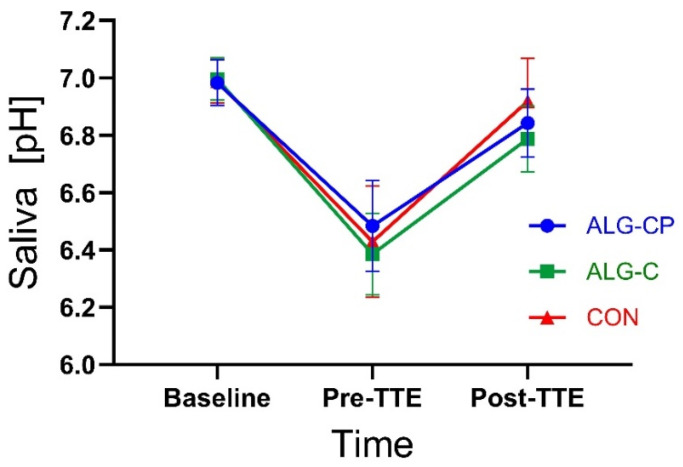
pH values of saliva during the clinical trial. Data are mean ± SEM.

There was a significant main effect of time in U-Creatinine (*p* = 0.02), with a significant increase in concentrations from baseline to post-TTE for ALG-CP and CON (*p* = 0.02 and *p* = 0.01, respectively) (Figure 8). No treatment effects were observed (*p* = 0.2). There were no time nor treatment effects for U-Carbamide levels (*p* = 0.6 and *p* = 0.3, respectively). Three participants had a higher level of U-Carbamide (>400 mmol/L) at baseline before ingesting ALG-C. Salivary pH was similar during the trial for all three supplements with no treatment effect found (*p* = 0.7, Figure 9). There was a main effect of time (*p* = 0.0001) with a drop in pH from baseline to pre-TTE and a rise again at post-TTE.

### 3.3. Gastrointestinal Responses

**Figure 10:**
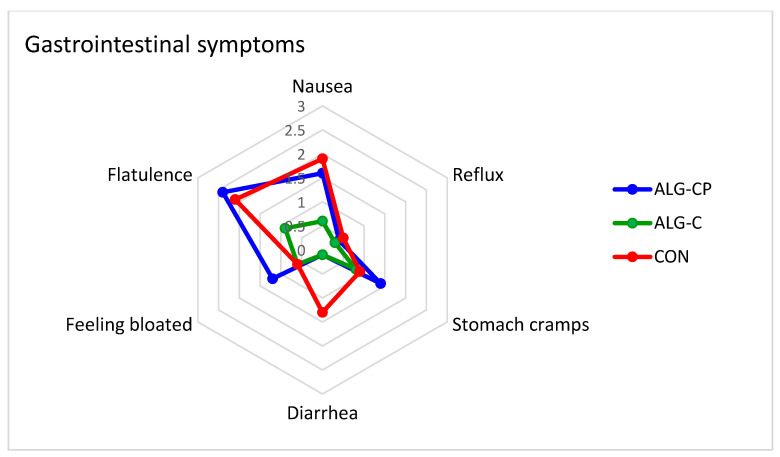
Radar chart of perceived gastrointestinal symptoms. Data are mean ± SEM.

An overview of the number of participants experiencing GI symptoms is presented in Table 1. Overall, most participants experienced absent-to-mild levels of GI symptoms. Some participants complained about GI symptoms when ingesting ALG-C compared to ALG-CP and CON. Five participants experienced severe GI symptoms (i.e., ≥5 on VAS [40]) in this study. This was particularly in regard to flatulence (*p* = 0.037), which was less evident in the ALG-C group (Figure 10). There were no further significant differences found between any of the groups for individual symptoms.

## 4. Discussion

The main objective of the present study was to compare the effect of a carbohydrate (CHO) hydrogel with (ALG-CP) or without (ALG-C) branched-chain amino acids and a CHO-only non-hydrogel (CON) on cycling performance. The current study is novel for two reasons. First, CHO was encapsulated in an alginate hydrogel prior to ingestion. Whereas previous investigations have only administered CHO-containing beverages with alginate, which forms a hydrogel, encapsulating CHO, when reaching the low pH of the stomach. Second, BCAA was added to one of the CHO-containing alginate hydrogels. To the best of our knowledge this is the first time a CHO-BCAA hydrogel has been investigated when ingested during cycling exercise.

In the present study, the participants cycled on average 65.28 ± 2.8 min with ALG-CP, 56.46 ± 10.92 min with ALG-C, and 59.89 ± 11.89 min with CON. Corresponding to a 29.6% and 29.1% longer cycling time with ALG-CP compared to ALG-C and CON. However, this was not statistically different between supplements. Previous studies have demonstrated that adding protein to a CHO-containing supplement improves performance after a recovery period of 5 to 18 h compared to CHO-only [14,15,16,17]. The addition of protein to a CHO supplement during exercise have in some studies shown an enhanced TTE performance compared to CHO alone, with improvements ranging between 13% and 40% [19,20,21,22]. While other investigations found no further benefits to performance [18,24,25,26,27,28]. These discrepancies may be due to differences in protocol designs across studies, including exercise intensity and duration and performance test.

In cycling, most races comprise periods of relative steady state as well as of high intensity work and/or sprinting [29]. For that reason, we designed a study where participants initially cycled for 2 h at various intensities. During this standardised cycling bout, participants ingested the CHO-containing supplements with or without the addition of BCAAs before the performance test. Previous studies [21,22] with similar designs to the present study, in which participants cycled for 3 h at varying intensities followed by TTE performance testing, reported better performances with CHO and protein ingestion compared to CHO only. In contrast, a study by Breen et al. [56], conducting a 2 h steady-state cycling bout at 50% Watt_max_ followed by a time-trial (TT) performance, reported similar performance when ingesting CHO with or without protein. This is in agreement with our findings.

In the current study, there was a lower heart rate (HR) during the standardised 2 h cycling bout (EX1) when ingesting ALG-CP compared to both CHO-only interventions, suggesting the cycling bout was less stressful with ALG-CP. The discrepancies in performance outcomes across studies may be due to the different performance tests included, as some conducted a time-to-exhaustion [18,19,20,21,22,24,26,28] and others a time-trial test [25,27,56]. In addition, some studies included an exercise bout before the performance test at moderate intensities (i.e., 60–70% VO_2max_), while others with varying intensities [18,21,22,24]. Additionally, some studies did not include a prior exercise bout, and the subjects had to complete a cycling bout at a specific intensity until fatigue [19,20,26,28] or a time trial [25]. Thus, differences in protocols between studies may have had an impact on the outcome. Finally, it is also possible that protein supplementation has a minimal effect on performance, which may be challenging to detect in certain study designs.

Adding BCAA to a CHO-containing supplement has been proposed to improve performance by potentially reducing muscle damage [12,18,19,20,57]. It has been hypothesised that BCAA supplementation may downregulate the secondary muscle damage response by increasing the bioavailability of amino acids Kenji et al. [58]. Specifically, it is suggested that BCAAs enhance glutamine synthesis through the transamination of glutamate, which subsequently influences the transcription of the nuclear factor of activated T-cells, thereby mitigating local inflammation Kenji et al. [58]. Moreover, significantly lower levels of granulocyte elastase (GEL) have been observed in the BCAA condition compared to the placebo condition, indicating a suppression of elevated inflammatory responses during exercise-induced muscle damage Kenji et al. [58]. Additionally, a significant reduction in the percentage of monocytes was noted in the BCAA condition, as these cells are known to differentiate into macrophages, which, if elevated, could further exacerbate the inflammatory response (Kenji et al. [58]).

In this study, there was a lower level of myoglobin before the start of the performance test when ingesting the CHO hydrogel with BCAA (ALG-CP) compared to CHO-only hydrogel (ALG-C). A study by Saunders et al. [19] demonstrated an 83% lower concentration of CK (as a marker of muscle damage) after an exhaustive exercise bout with protein added to a CHO supplementation compared to CHO-only. This was accompanied by a substantial longer time-to-exhaustion performance [19]. Similarly, Romano-Ely et al. [26] observed significantly lower levels of muscle damage markers (CK and LDH) at 24 and 72 h after the first exercise bout with intake of CHO and protein compared to CHO only. However, their study did not show an improvement in TTE performance with the combined intake of CHO and protein relative to CHO alone [26]. In the present study, we report a main effect of time on all muscle damage markers, with an increase observed throughout the exercise protocol. Additionally, we noted significantly lower plasma myoglobin levels immediately before the onset of TTE performance in the ALG-CP condition compared to the ALG-C condition, suggesting a reduction in muscle damage associated with ALG-CP supplementation. No further differences were observed between supplements in muscle damage markers. Moreover, we did not measure the levels of muscle damage markers during the 2 h recovery nor 24 or 72 h post-exercise [26] and, thus, cannot elucidate whether a potential difference may have occurred between supplements during the recovery phase. In the present study, the participants cycled, on average, 29.6% longer, when ingesting ALG-CP compared ALG-C. However, it was not statistically significant and we cannot associate the enhanced performance with improved muscle damage in the current study, improved muscle damage may still partly help maintain or improve performance [18,19,20,26].

In this study, we observed an increase in insulin levels with the intake of ALG-CP during the first phase of exercise compared to both CHO conditions, which is in contrast to most prior studies demonstrating no differences in insulin levels during exercise when ingesting CHO with or without protein [18,21,22,24,25]. Previous studies have demonstrated that specific AAs added to a CHO beverage can induce a higher insulin response during rest or recovery compared to CHO only [16,59,60,61,62,63]. Nonetheless, the observed increase in insulin levels with ALG-CP during exercise is within the range of 20–100 pmol/L, which is much lower than the insulin levels shown during recovery with combined CHO and PRO intake (300–400 pmol/L) [7,8,14,60,61,63]. Furthermore, in this study the glucagon levels remained constant during exercise (until pre-TTE) when participants were provided with the CHO-containing supplements. Importantly, CHO ingestion maintained glucose at about 5 mM and the glucagon levels did not increase [6,63,64]. Interestingly, before initiating the TTE performance test, there was a higher glucagon level with ALG-CP compared to both CHO-only interventions, which could potentially be due to an increase in BCAA availability in the circulation, which has been shown to stimulate glucagon secretion [62].

The second aim of the study was to compare two different delivery systems of CHOs during exercise (i.e., hydrogel vs. non-hydrogel). The benefits of the alginate delivery system has been proposed to allow CHO consumption in large amounts, increasing the gastric emptying (GE) rate and reducing incidence of GI distress, which could lead to an improved performance [43,46,48,49]. In this study, we provided 75 g CHO/h during exercise, which is within the range of CHO administered in prior studies (i.e., 68–95 g/h) and within the recommendations for optimising performance [9,43,47,49,53,54]. Additionally, CHO consumption appear to be related to cycling performance in a curvilinear dose–response manner, with optimal performance achieved with a CHO intake rate of approximately 78 g of CHO/hour when cycling for 2 h [5,65]. Similarly to previous studies on hydrogels, we matched the supplements on CHO content, ensuring the subjects ingest optimal amounts of CHO during prolonged exercise [9,49,53,54,66]. In this study, we found comparable levels of plasma glucose between supplements during the exercise bout, similar to previous findings in both thermoneutral and hot–humid conditions [48,49,53,54,66]. Moreover, in line with previous studies, we report similar cycling performances when adding alginate to a CHO supplement compared to CHO only [47,53,54]. Baur et al. [53] demonstrated similar cycling sprint performances when ingesting alginate CHO compared to a CHO-only beverage. Likewise, McCubbin et al. [54] demonstrated similar TTE running performances comparing a CHO hydrogel with a CHO non-hydrogel. Furthermore, studies implementing a TT performance test also found no significant differences in performances [47,66,67]. A more recent study by Rowe et al. [49] did, however, report a 2.1% enhanced 5 km TT running time with a CHO hydrogel. CHO contribution to total energy expenditure is increased with increasing intensity [68,69]. Thus far, studies investigating the effects of alginate-based CHO hydrogels on performance capacity have utilised a wide spectrum of exercise intensities [47,49,53,54]. Some studies have focused on lower-intensity exercise (e.g., ≤60% VO_2max_) [43,47,54,66], while others included moderate exercise intensities (e.g., 69% VO_2max_) [49,67]. These variations in exercise protocols highlight the need to better understand how different intensities influence the efficacy of alginate-based CHO supplementation.

Although intake of CHO in high concentrations during exercise may improve performance, it may also cause GI distress, which has been associated with impaired athletic performance [30,34,36,37,40]. In this study, participants experienced absent-to-mild GI symptoms (<5 on VAS), which is similar to the reported symptoms of prior studies [43,53,54]. Consistent with McCubbin et al. [54], the incidence of severe GI symptoms was lower with the CHO-only hydrogel, especially in regard to flatulence (*p* = 0.037). However, the rating of GI issues perceived during the trial was generally low in the control group as well (Table 1), which may mask a potential positive effect of the hydrogel. Another aspect of CHO supplementation is the potential risk of deteriorating dental health. Due to the intake of CHO in large quantities, athletes are considered at risk of developing carious lesions [41,42,43], which form as a result of a process in which tooth demineralisation occurs due to organic acid (e.g., lactic acid) being produced by bacterial degradation of sugars [44,45]. In this study, we measured the pH of saliva as an indirect method to detect the potential risk of developing carious lesions. We report a similar pH profile between all three CHO-containing supplements with a drop in pH at pre-TTE compared to baseline and a recovery in pH again at post-TTE (Figure 9). The drop in pH was within the normal pH range [70].

### Strengths and Limitations

A strength of this clinical trial is that it is conducted as a randomised, double blinded and cross-over controlled trial, where subjects were their own controls. In addition, only subjects that were already accustomed to cycle training were recruited, thereby minimising the risk of learning effect influencing the outcome. A familiarisation protocol was conducted prior to the experimental days, reducing a potential learning and practice effect. We provided standardised dinner and breakfast before each experimental day. Although study participants cycled on average (calculated per participant) 29.6% and 29.1% longer after intake of ALG-CP in comparison to ALG-C and CON, respectively, no statistically significant differences were observed. Performance improvements of this magnitude are meaningful in the context of professional competitive cycling.

Prior studies have indicated significant improvements in TTE, ranging from 13% to 40% with combined CHO and protein ingestion compared to CHO alone [19,20,21,22]. We noted a relatively high variability in cycling duration during TTE among participants, a factor that might have been addressed by enrolling more participants. However, a previous study has shown significantly better TTE performance in cyclists with nine participants after intake of CHO and protein during exercise [21]. Recruiting both triathletes and cyclists provided a sufficient sample size, yet focusing solely on cyclists, for instance, could have reduced data variability. A limitation of the present study is that we did not include muscle biopsies to help explain the differences in the recovery period. Furthermore, we did not measure AA plasma levels, which may have helped explain the metabolic responses to the interventions. The assessment of dental health and GI symptoms were acute and subjective for the GI evaluation, and for future investigations a more long-term evaluation of CHO hydrogels on dental health would be of interest.

## 5. Conclusions

The participants cycled on average ~8.8 min and ~5.4 min longer during TTE, when ingesting ALG-CP compared to ALG-C and CON, respectively. While ALG-CP improved performance duration compared to ALG-C and CON, the difference did not reach statistical significance. The lower HR during the cycling session with ALG-CP suggests that it was less stressful compared to both CHO supplements. Additionally, there was a significant lower level of myoglobin with ALG-CP before initiating the performance test compared to ALG-C, indicating participants being in a better nutritional state with less muscle damage when consuming ALG-CP. There were no differences in pH of saliva between supplements. Most GI symptoms ranged from *none to mild* in this study, some subjects perceived severe GI symptoms (>5 on VAS). This was particularly in regard to nausea and flatulence. These findings suggest ALG-CP may offer advantages in cycling performance and recovery.

## Figures and Tables

**Table 1:** Gastrointestinal symptoms.

	ALG-CP		ALG-C		CON	
Symptoms	N	M ± SEM	N	M ± SEM	N	M ± SEM
Nausea	4	1.6 ± 0.9	3	0.6 ± 1.1	5	1.9 ± 0.8
Flatulence	8	2.4 ± 0.7	4	0.9 ± 0.4	7	2.1 ± 0.8
Reflux	1	0.4 ± 0.4	2	0.3 ± 0.2	2	0.5 ± 0.4
Stomach cramps	5	1.4 ± 0.7	3	0.8 ± 0.5	4	0.9 ± 0.5
Feeling bloated	4	1.2 ± 0.6	3	0.6 ± 0.4	4	0.6 ± 0.3
Diarrhoea	1	0.1 ± 0.1	1	0.1 ± 0.1	3	1.3 ± 0.8

Numbers (N) of participants experiencing GI symptoms. Mean (M) ± SEM are given for each supplement. N = 10 participants.

## Data Availability

Data are contained within the article.
